# Characterization of Novel Selected Microalgae for Antioxidant Activity and Polyphenols, Amino Acids, and Carbohydrates

**DOI:** 10.3390/md20010040

**Published:** 2021-12-30

**Authors:** Paula Santiago-Díaz, Argimiro Rivero, Milagros Rico, Juan Luis Gómez-Pinchetti

**Affiliations:** 1 Departamento de Química, Universidad de Las Palmas de Gran Canaria, Campus de Tafira, 35017 Las Palmas de Gran Canaria, Spain; paula.santiago@ulpgc.es (P.S.-D.); argimiro.rivero@ulpgc.es (A.R.); 2 Instituto de Oceanografía y Cambio Global (IOCAG), Campus de Taliarte, Universidad de Las Palmas de Gran Canaria, Unidad Asociada ULPGC-CSIC, 35214 Telde, Spain; juan.gomez@ulpgc.es; 3 Banco Español de Algas (BEA), Universidad de Las Palmas de Gran Canaria, Muelle de Taliarte s/n, 35214 Telde, Spain

**Keywords:** amino acids, carbohydrates, microalgae, phenolic compounds, radical scavenging activity (RSA), RP-HPLC

## Abstract

The biochemical composition of three novel selected microalgae strains (Chlorophyta) was evaluated to confirm their potential possibilities as new sustainably produced biomass with nutritional, functional, and/or biomedical properties. Extracts from cultured *Pseudopediastrum boryanum*, *Chloromonas cf. reticulata*, and *Chloroidium saccharophilum* exhibited higher radical scavenging activity of DPPH (1,1-diphenyl-2-picrylhydrazyl) when compared to butylated hydroxytoluene (BHT), but lower than butylated hydroxyanisole (BHA). Total phenolic compounds and amino acids were determined by newly developed RP-HPLC methods. Total phenolic contents, as µg g^−1^ of dry biomass, reached 27.1 for *C. cf. reticulata*, 26.4 for *P. boryanum*, and 55.8 for *C. saccharophilum*. Percentages of total analysed amino acids were 24.3, 32.1, and 18.5% of dry biomass, respectively, presenting high values for essential amino acids reaching 54.1, 72.6, and 61.2%, respectively. Glutamic acid was the most abundant free amino acid in all microalgae samples, followed by proline and lysine in *C. saccharophilum* and *P. boryanum*, and methionine and lysine in *C. reticulata*. Soluble carbohydrates in aqueous extracts ranged from 39.6 for *C. saccharophilum* to 49.3% for *C. reticulata*, increasing values to 45.1 for *C. saccharophilum* and 52.7% for *P. boryanum* in acid hydrolysates of dried biomass. Results confirmed the potential possibilities of these microalgae strains.

## 1. Introduction

Changes in human and animal nutrition are essential, among other actions, to achieve several of the UN Sustainable Development Goals [1]. Diets rich in meat and processed foods are detrimental to health and are also associated with higher environmental costs and greenhouse gas emissions. Therefore, it is a priority to reduce their consumption in order to mitigate their negative impact on health [2].

Algae in general and microalgae in particular are described as a novel rich source of nutrients and contain natural products with several properties and applications in many industrial fields, including food, feed, cosmetics, pharmaceuticals, and biofuel production [3,4,5]. 

Polysaccharides from macro- and microalgae are considered a source of dietary fibre with bioactive properties improving the levels of blood glucose and cholesterol [6,7]. These algal polysaccharides also show other potent biological activities such as antioxidant, antifungal, antiviral, antibacterial, and antitumoral properties; tyrosinase inhibitory activity; and anti-inflammatory and immunomodulatory characteristics [6,8]. 

Balanced diets in amino acids of natural origin and safe sources are strongly recommended [9]. Proteins are one of the main components of microalgae, reaching up to 70% of dry biomass in some species [10,11] and containing up to 50% essential amino acids and higher antioxidant activities than those of common proteins in the human diet [12,13]. Several microalgal peptides also exhibit antihypertensive, immunomodulatory, anticancer, hepatoprotective, antiatherosclerosis, anticoagulant, anti-UV radiation, antiosteoporosis, and antimicrobial activities [14]. Studies focused on drugs combined with glutamic acid (glutamate) confirmed an increase in their efficacy [15]. In addition, glutamic and aspartic acids contribute to enhancing (i) the flavour of meat, soy sauce, seafood, and some processed foods [16] and to (ii) protein solubility for pharmaceutical uses [17]. Dietary supplementation with proline may also be advantageous under certain physiological and pathological conditions [18].

In addition, different types of antioxidants such as phenolic compounds from seaweeds and microalgae have also been reported [19,20,21]. Food enrichment with microalgae is a simple and well-known method for improving the physicochemical, nutritional, and sensory properties [22]. The antioxidant capacity and phenolic content of broccoli soup increased when freeze-dried *Spirulina* sp., *Chlorella* sp., or *Tetraselmis* sp. was added at concentrations ranging from 0.5 to 2.0% (*w/v*) [23]. According to Žugčić et al. [24], beef patties prepared with microalgal proteins (1% *Chlorella* or 1% *Spirulina* of 60 and 70% purity, respectively) increased the concentrations of all amino acids, especially aspartic and glutamic acids, concluding that microalgae proteins could be useful candidates for new meat products in the food and feed industries [25].

Considering all this application potential and despite the rich biodiversity of microalgae, only a few species are exploited from a biotechnological point of view, and only 18 species of the phylum Chlorophyta are being produced in Europe [26]. The objective of this study was to evaluate the biochemical composition of three selected, not previously studied, freshwater microalgae strains *Chloromonas cf. reticulata, Pseudopediastrum boryanum*, and *Chloroidium saccharophilum* for their potential in developing food and feed products with high nutritional and therapeutic/functional values. For this purpose, methanol extracts obtained from laboratory-controlled cultured biomass were screened for their capacity to scavenge the 1,1-diphenyl-2-picrylhydrazyl (DPPH) radical and compared with food additives (butylated hydroxyanisole (BHA) and butylated hydroxytoluene (BHT)) with known antioxidant activities [27]. Ten different phenolic compounds and ten selected amino acids were identified and quantified by newly developed RP-HPLC methods, and total soluble carbohydrate contents obtained by two different extraction protocols were also determined.

## 2. Results

### 2.1. Radical Scavenging Activity

**Figure 1:**
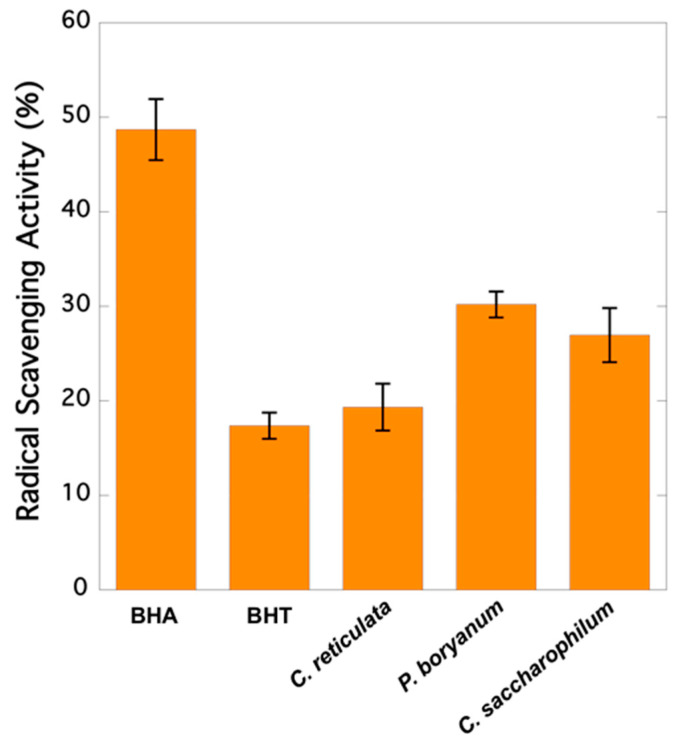
Radical scavenging activities (RSA) of synthetic compounds (BHA and BHT) and microalgae extracts expressed as DPPH inhibition percentage: 100 × (1 − Abs in the presence of sample/Abs in the absence of sample).

As it is observed in Figure 1, extracts from *Pseudopediastrum boryanum* exhibited the highest capacity to scavenge free radical DPPH (30.19%), followed by *Chloroidium saccharophilum* (26.95%) and *Chloromonas cf. reticulata* (19.33%). All microalgae samples showed a higher RSA than BHT (17.37%) and lower activity than BHA (48.69%).

### 2.2. Identification and Quantification of Phenolic Compounds

The identification and quantification of 10 polyphenols were achieved by an updated RP-HPLC method in less than 30 min (retention times ranged from 4.69 to 25.87 min). Linearity was evaluated using the method of least squares of a plot of integrated peak area versus mean concentration from three area measurements. The correlation coefficients were not less than 0.9995. Precision was assessed using six determinations at 1 µg mL^−1^ and expressed as relative standard deviation (RSD), which ranged from 1.80 to 3.85%. The limits of detection (LOD) and the limits of quantification (LOQ) were calculated assuming a minimum detectable signal-to-noise level of 3 and 10, respectively. LOD were found to be in the range of 0.0221–0.2003 µg mL^−1^, and the LOQ were observed in the range of 0.0736–0.6676 µg mL^−1^. The recoveries were found in the range from 91.8 to 109.2%. 

All ten analysed phenolic compounds were identified for the strain *Chloroidium saccharophilum* (Table 1). Rutin and protocatechuic, coumaric, ferulic, and gentisic acids were not detected in the extracts obtained from *Chloromonas reticulata. Pseudopediastrum boryanum* showed a lack of coumaric and gentisic acids. *C. saccharophilum* exhibited the highest content of phenolic compounds (55.83 µg g^−1^ of dry weight) followed by *C. reticulata* and *P. boryanum* (27.10 and 26.40 µg g^−1^ of dry weight), respectively.

### 2.3. Identification and Quantification of Free and Total Analysed Amino Acids

A new RP-HPLC method was developed for detecting and quantifying 10 amino acids in microalgae extracts in less than 35 min (the retention times ranged from 6.19 to 34.87 min). Correlation coefficients were not lower than 0.9976. Precision expressed as RSD, the LODs, and the LOQs were calculated as above. RSD ranged from 1.73 to 3.88%. The percentage recoveries were from 92.4 to 101.8%. The LOD ranged from 0.0006 to 0.01 µg mL^−1^, and the LOQ from 0.0015 to 0.0335 µg mL^−1^. This methodology was fast, precise, and accurate and allowed the simultaneous quantification of 10 amino acids with a high sensitivity and reproducibility.

All 10 free amino acids studied were present in all the microalgae strains (Table 2). Glutamic acid was the most abundant in all microalgae samples, ranging from 461.82 to 5630.37 µg g^−1^ of dry weight. *C. saccharophilum* exhibited a remarkably higher amount of each single amino acid compared to the other microalgae strains, reaching 20.46 mg of total free amino acids per gram of dry biomass.

Apart from values for glutamic acid, Table 2 shows that the most abundant free amino acids in *C. saccharophilum* were proline, lysine, valine, and phenylalanine; in *P. boryanum* were proline, lysine, and arginine; and in *C. reticulata* were lysine and methionine. The percentages of essential amino acids were 49.7% in *C. reticulata*, 38.2% in *C. saccharophilum*, and 28.6% in *P. boryanum* (Table 2). 

Table 3 shows the total content of the ten amino acids analysed in the acid-hydrolysed microalgae extracts. Total contents of these amino acids in *P. boryanum*, *C. reticulata*, and *C. saccharophilum* were 32.06, 24.27, and 18.45% on a dry weight basis, with high percentages of the analysed essential amino acids: 72.6, 54.1, and 61.2%, respectively. Lysine was the most abundant amino acid in *C. saccharophilum* (36.49 mg g^−1^ of dry weight)*,* proline in *C. reticulata* (51.69 mg g^−1^ of dry weight), and methionine in *P. boryanum* (137.20 mg g^−1^ of dry weight).

### 2.4. Carbohydrate Contents

**Figure 2:**
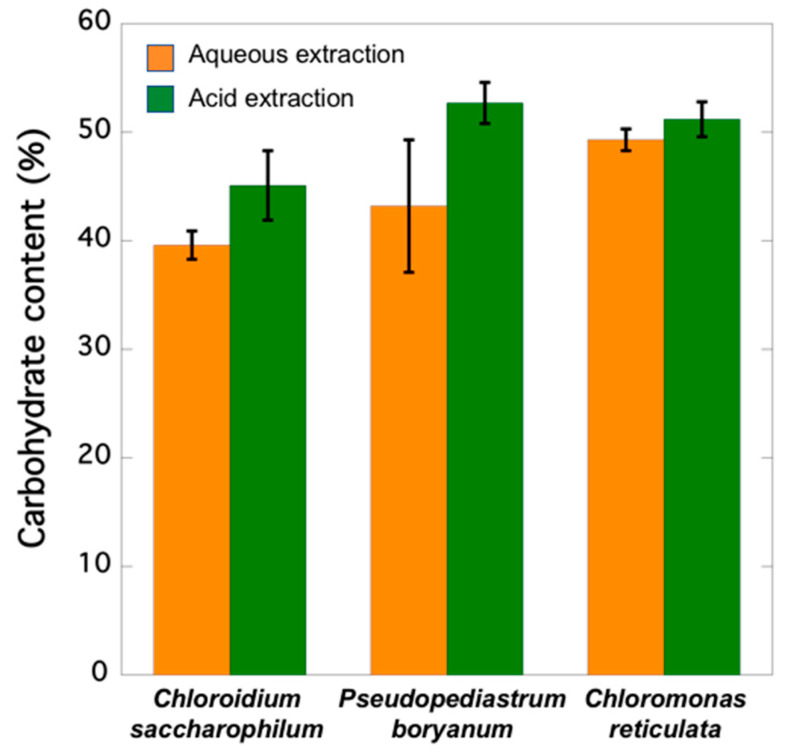
Carbohydrate content (as percent of glucose equivalents per dry weight) of the analysed microalgae strains.

Aqueous extraction of *C. saccharophilum, P. boryanum, and C. reticulata* biomass showed 39.64, 43.24, and 49.25% in soluble carbohydrates, respectively (Figure 2). The highest carbohydrate contents reaching 45.06, 52.67, and 51.24% were obtained when extractions were carried out under acid hydrolysis conditions.

## 3. Discussion

### 3.1. Algal Material and Extraction Procedures

The Canary Islands are mountainous with a sub-tropical volcanic origin, supporting high levels of solar radiation all year round. These environmental conditions generate highly diverse habitats and ecosystems, forcing microorganisms to adapt and accumulate metabolites that might be interesting from a biotechnological approach [28].

The selected microalgae strains analysed in the present study were bioprospected from different locations and environments and, after clonal isolation, adapted to laboratory growth conditions and BG-11 culture media before scale-up for the evaluation of growth characteristics and biomass production.

Several factors should be considered in the extraction and quantification of metabolites, such as the previous treatment of microalgae cells and storage, the hydrolysis conditions (time, temperature, and acid concentration), and the mechanical/chemical extraction methods. Cell wall disruption is a necessary preliminary step to make the cell contents accessible and digestible and prevent incorrect measurements. The efficiency of cell disruption methods depends on the species being investigated (including cell wall type) and their physiological state. Therefore, the total rupture of the cell membrane should be confirmed by making observations with a microscope to prevent underestimation of the metabolite contents [11].

Kröger et al. [29] compared several methods for effective extraction from the microalgae *Scenedesmus rubescens*, concluding that freeze-drying produces cell wall damage and therefore improves the extraction yields. Moreover, several drying methods have also been studied by de Farias Neves et al. [30], who confirmed that freeze-drying is the most suitable microalgae drying method without bioactive compound loss. For all these reasons, in our work, biomass was freeze-dried and cells examined under a microscope to check complete cell wall breaking.

### 3.2. Antioxidant (RSA) Activity and Phenolic Contents

The synthetic compounds BHA and BHT are widely used as antioxidant food additives at a maximum level of 0.02% [31]. However, these compounds are classified as cancer promoters capable of inducing cytotoxicity and apoptosis [32]. Therefore, many studies have extensively studied their replacement by safer and inexpensive natural antioxidants. Phenolic compounds of natural origin exhibit antioxidant properties capable of extending food shelf life by preventing rancidity due to oxidation. They also exhibit protective effects against oxidative stress in biological systems [19]. 

Previous studies showed that gentisic acid, gallic acid, catequin, epicatechin, protocatechuic, and syringic acids exhibited higher antioxidant activity than BHT and BHA [33]. Catechin, epicatechin, gallic acid, and vanillic acid, among others, are better radical scavengers than many monomeric flavones and even flavonols because they act particularly well as H-atom donors [34]. The presence of these compounds in microalgae increases their potential health benefits. The microalgae species *C. saccharophilum, P. boryanum*, and *C. reticulata* showed a higher RSA than that of BHT (Figure 1). Gentisic and coumaric acids were not detected in the extracts derived from *P. boryanum* (Table 1), which showed the highest RSA (30.19%) and the lowest phenolic content (26.40 µg g^−1^ of dry biomass). All ten phenolic compounds, gallic acid, protocatechuic acid, catechin, vanillic acid, rutin, epicatechin, syringic acid, gentisic acid, coumaric acid, and ferulic acid, were detected in *C. saccharophilum*, which exhibited the highest content of phenolic compounds, followed by *C. reticulata* and *P. boryanum* (55.83, 27.10, and 26.40 µg g^−1^ of dry biomass, respectively). The lack of five analysed phenolics (rutin and coumaric, ferulic, protocatechuic, and gentisic acids) was observed in the extracts prepared with *C. reticulata*, which gave the lowest relative RSA (19.33%), but a phenolic content similar to that of *P. boryanum* (Table 1). Similar findings were reported by Corrêa da Silva et al. [35] who studied the total phenolic content and antioxidant activity of microalgae *P. boryanum* grown in different culture media. Their results also showed extracts with higher phenolic content (measured through the Folin–Ciocalteu assay) but lower antioxidant activity (assay ABTS (2,2′-azino-bis(3-ethylbenzothiazoline-6-sulphonic acid)). No correlation between DPPH inhibition and phenolic contents was found by Blagojević et al. [36], who evaluated the phenolic profiles and antioxidant activities of 10 cyanobacterial strains cultured in BG11 medium in the presence and absence of nitrogen. In our study, a small group of phenolic compounds was quantified. The antioxidant response of these compounds varies remarkably depending on their chemical structure [34], and other possible antioxidants present in the mixture have not been considered, as phenolics are not the only contributors to the antioxidant activities in algae. In addition, the extracts are complex mixtures including active components at low levels, and their activities depend on the relative concentrations of these components and the interfering compounds as well as the synergistic, additive, or antagonistic effects between them [37]. 

Samples in this study showed a higher content of phenolic compounds than five different microalgae and cyanobacterial species evaluated by Klejdus et al. [38], who identified eight phenolic compounds and quantified the highest content in the green microalgae *Spongiochloris spongiosa* (6.656 µg g^−1^ of dry biomass). Blagojević et al. [36] only detected 8 of 45 polyphenols investigated in several cyanobacteria species. These authors observed that cyanobacteria *Arthrospira* S1 and *Anabaena* C2 showed a lower total phenolic content (24.05 and 18.72 µg g^−1^ of biomass, respectively) than those obtained in our samples. Onofrejová et al. [39] identified 12 phenolic compounds in the freshwater microalgae *Spongiochloris spongiosa* and cyanobacterium *Anabela doliolum*, also quantifying lower contents (5.1 and 3.6 µg g^−1^, respectively) than those determined in the present study.

### 3.3. Free Amino Acid Contents

In this study, 10 amino acids were selected because of their antioxidant properties and their important role in cellular metabolism as key precursors for synthesis of several metabolites [9,40,41,42,43]. Therefore, a well-balanced diet can ensure the intake needs of essential and non-essential amino acids of the body to function properly.

All microalgal samples presented the 10 free amino acids evaluated—histidine, methionine, valine, lysine, isoleucine, phenylalanine, arginine, proline, and glutamic and aspartic acids—with quantitative differences for individual compounds between the three different strains (Table 2). Glutamic acid was the most abundant, ranging from 461.8 to 5630.4 µg g^−1^ of dry biomass. These results are in accordance with those reported by Vendruscolo et al. [44], who quantified 15 amino acids in two Chlorophyceae (*Chlorella vulgaris* and *Scenedesmus obliquus*) and two cyanobacteria (*Aphanothece microscopica* and *Phormidium autumnale*), concluding that glutamic acid was the most abundant detected amino acid in three of them (*Scenedesmus obliquus* showed a higher content of alanine). Our findings also agree partially with previous studies focused on the free amino acid profile determination of seaweeds and microalgae, which confirmed that glutamic and aspartic acids were the most abundant free amino acids (up to 26% of the free amino acid fraction) [45,46].

*C. saccharophilum* showed the highest amount of total analysed free amino acids, reaching 20.5 mg g^−1^ of dry biomass (13.34 and 3.82 times higher than those of *C. reticulata* and *P. boryanum*, respectively). Vendruscolo et al. [44] reported lower amounts of the total sum of 15 amino acids quantified in two Chlorophyceae and two cyanobacteria (ranging from 0.371 to 1.525 mg g^−1^ of dry biomass). Machado et al. [47] determined the total sum of 20 free amino acids in four seaweed species (*Porphyra dioica*, *Porphyra umbilicalis*, *Gracilaria vermiculophylla*, and *Ulva rigida)*, which ranged from 3.36 to 16.17 mg g^−1^ of dry biomass. Their values were higher than those for *C. reticulata,* but lower than those found for *C. saccharophilum* in this study (1.53 mg and 20.46 mg g^−1^ of dry biomass, respectively).

Apart from glutamic acid, Table 2 shows that the most abundant free amino acids were proline, lysine, valine, and phenylalanine in *C. saccharophilum*; proline, lysine, and arginine in *P. boryanum*; and lysine and methionine in *C. reticulata*. On the contrary, Vendruscolo et al. [44] only detected methionine and lysine in one of the four analysed microalgae (30.55 and 8.05 µg g^−1^ of dry biomass, respectively, in *Scenedesmus obliquus*), and Machado et al. [47] only identified methionine in one of the above cited red seaweed (160 µg g^−^^1^ of dry biomass in *Porphyra dioica*). 

Under natural conditions, the composition of free amino acids in microalgae can vary dramatically during the growing season. Kolmakova et al. [45] concluded that the composition of free amino acids of diatoms and green microalgae and cyanobacteria is extremely sensitive to external factors such as available nitrogen and light intensity and photoperiod and also depends on the growth phase of the microalgae culture [48]. According to Granum et al. [49], extracellular free amino acid contents exuded by the marine diatom *Skeletonema costatum* changed drastically from the exponential to stationary growth phase, and the cellular free amino acid levels reached values between 8% (end of scotophase) and 22% (end of photophase) of cellular organic N and decreased by 90% within 24 h of N depletion. In fact, intracellular amino acids have been proposed to be used as an index of the physiological status of the diatom *Rhizosolenia delicatula* [50].

### 3.4. Total Contents of Analysed Amino Acids

Kolmakova et al. [45] reported that the percentages of total essential and non-essential amino acids in diatoms and green microalgae and cyanobacteria are stable and show a common profile, with leucine as the most abundant essential amino acid, methionine and histidine as the least abundant, and glutamic and aspartic acids as the most abundant non-essential amino acids (up to 20% of the sum of amino acids). However, methionine was the most abundant amino acid in *P. boryanum* in the present study (137.2 mg g^−1^ of dry weight). Several authors have confirmed that non-essential glutamic and aspartic acids in the cell hydrolysates constituted 22–44% of the total amino acids in algae [51]. Cobos et al. [52] reported relatively similar amino acid profiles in four freshwater Chlorophyta microalgae, *Ankistrodesmus* sp., *Haematococcus* sp., *Scenedesmus* sp., and *Chlorella* sp., where aspartic acid ranged from 20.94 to 38.21 mg g^−1^ of dry weight and leucine from 20.08 to 40.99 mg g^−1^ of dry weight, and the least abundant amino acid was histidine, ranging from 4.10 to 7.24 mg g^−1^ of dry weight. The percentages of glutamic and aspartic acids in this work were 9.6, 15.9, and 18.4% in *P. boryanum*, *C. reticulata*, and *C. saccharophilum*, respectively, which would presumably decrease if a larger number of amino acids were analysed. In accordance with Cobos et al. [52] and Kolmakova et al. [45], the least abundant amino acid was histidine, ranging from 1.81 to 6.07 mg g^−1^ of dry weight (Table 3). However, methionine, proline, and lysine were the most abundant amino acids in *C. reticulata* and *P. boryanum*, while *C. saccharophilum* gave a higher content of lysine, proline, and glutamic acid. Lysine was the most abundant amino acid in *C. saccharophilum* (36.49 mg g^−1^ of dry weight) and proline in *C. reticulata* (51.69 mg g^−1^ of dry weight). Several studies have also exhibited relevant amounts of lysine and proline [11,53]. *C. saccharophilum,* which showed the highest free amino acid content, presented the lowest total content of analysed amino acids after acid hydrolysis, followed by *C. reticulata* and *P. boryanum* (184.5, 242.7, and 320.6 mg g^−1^ of dry weight, respectively). The analysis of total amino acids does not distinguish between free amino acids, which represent less than 10% of the total amino acids, and those which are bound in proteins [54]. This fact can lead to significant differences between free and total amino acid profiles. In addition, it is important to note that high total analysed amino acid values were obtained, considering that only 10 amino acids were quantified in the present study. Machado et al. [47] evaluated the presence of 20 amino acids in four macroalgae (*Porphyra dioica*, *Porphyra umbilicalis*, *Gracilaria vermiculophylla*, and *Ulva rigida*) and reported total contents of amino acids ranging from 96.22 to 286.56 mg g^−1^ of dry weight, and from 57.63 to 173.08 mg g^−1^ of dry weight if only the 10 amino acids analysed in our study were considered. These authors found fractions of free amino acids ranging from 3.15 to 7.18 g per 100 g of total amino acids and from 3.56 to 5.57% considering only our 10 amino acids. Vieira et al. [13] reported higher fractions of free amino acids (grams per 100 g of total amino acids) ranging from 6.47 to 24.0% in brown seaweed species and from 3.40 to 14.0% in red and green seaweeds. These differences may be due to the fact that the extraction of free amino acids was carried out with 0.2M perchloric acid, which could have hydrolysed peptides and increased the amount of free amino acids versus the aqueous extraction performed by Machado et al. [47]. Our results showed lower fractions of the 10 analysed free amino acids in *C. reticulata* and *P. boryanum* (0.63 and 1.67%, respectively) and a higher fraction in *C. saccharophilum* (11.1%) than those reported by Machado et al. [47]. 

Higher percentages of the analysed essential amino acids were found in *P. boryanum* and *C. saccharophilum* (72.6 and 61.2%, respectively) than those found by Machado et al. [46] when only the 10 amino acids evaluated in this study were considered (between 54% in *Gracilaria vermiculophylla* and 57.87% in *Ulva rigida*). *C. reticulata* showed a similar percentage of the analysed essential amino acids (54.1%). In accordance with Sui et al. [55], these high percentages could be due to the illumination cycle applied during the culture (18:6 h L:D) and the late-exponential growth phase for harvesting cells. Araya et al. [56] quantified the content of seventeen amino acids in five species of microalgae (*Haematococcus pluvialis*, *Skeletonema costatum*, *Arthrospira* sp., *Acutodesmus acuminatus*, and *Botryococcus braunii*). Their results showed that the highest amount of amino acids (267.6 mg g^−1^) was found in *Arthrospira* sp., and the other four species contained lower amounts than those found in this study (below 141.3 mg g^−1^ found in *Botryococcus braunii*).

Lourenço et al. [54] found different amino acid contents (as percentage of dry matter) in *Chlorella minutissima* and *Prorocentrum minimum* at the following growth phases: mid-exponential (24.79 and 24.18%, respectively), late-exponential (36.96 and 30.40%, respectively), early stationary (36.12 and 27.44%, respectively), and late-stationary (22.46 and 26.25, respectively). 

On the other hand, different microalgae species show specific needs of L:D cycles and light intensity for productive photosynthesis [57]. Long illumination periods have been previously reported as a stress condition to induce the accumulation of lipids and carotenoids such as astaxanthin [58]. Sui et al. [55] studied the impact of two L:D cycles (12:12 and 24:0 h) on *Dunaliella salina* protein production, concluding that continuous illumination led to higher protein content (0.62 g L^−1^ on day 16 in the exponential phase), which decreased in the stationary phase (0.49 g L^−1^ on day 28). On the contrary, microalgae cultured under a 12:12 h L:D cycle gave a constant accumulation of proteins that reached 0.43 g L^−1^ in the stationary phase. The contents of all individual essential amino acids increased between 5% and 58% in cells cultured under an L:D cycle, reaching 30% of the total protein, and increased dramatically by 17-125% in cells cultured under continuous illumination, reaching 44% of the total protein content. Seyfabadi et al. [59] studied the behaviour of *Chorella vulgaris* incubated at 37.5, 62.5, and 100 µmol photons m^−2^ s^−1^ irradiance and 8:16, 12:12, and 16:8 h L:D photoperiods. It was confirmed that a longer illumination period increases protein contents. In fact, the cycle 16:8 h L:D yielded the highest protein accumulation under each irradiance assayed, reaching the maximum at 100 µmol photons m^−2^s^−1^ irradiance and a 16:8 h L:D cycle. The photoperiod 16:8 h L:D used in the present study also might stimulate the production of several essential amino acids.

Gorissen et al. [60] analysed the amino acid contents after the acid hydrolysis of 35 protein samples commercially available as isolated protein powder suitable for application in human nutrition or animal feeds. *P. boryanum* showed a higher total amount of six essential amino acids than several of these dietary protein samples, whose content of eight essential amino acids was quantified (oat (137), lupin (131), wheat (180), hemp (116), microalgae (157), soy (199), brown rice (221), corn (210), and egg (165), where values in parentheses mean mg g^−1^ of raw material). *C. saccharophilum* and *C. reticulata* also showed comparable contents to several protein sources analysed by Gorissen et al. [60]. The high levels of the analysed amino acids with high percentages of essential amino acids make microalgae strains in our study a novel source of essential amino acids with potential use in food and functional products.

### 3.5. Carbohydrate Contents

*C. saccharophilum, C. reticulata*, and *P. boryanum* showed 45.1, 51.2, and 52.7% in carbohydrates, respectively (Figure 2). These results partially agree with those reported by Schulze et al. [61] who analysed freeze-dried biomass from 46 microalgae after hydrolysis with 2N HCl for 1 h and revealed carbohydrate contents ranging from 16.5% (*Mychonastes* sp.) to 71.6% (*Porphyridium purpureum*). Templenton et al. [62] published lower amounts of carbohydrates in strains of *Phaeodactylum tricornutum*, *Nannochloropsis* sp., and *Chlorella vulgaris* (19.6, 8.6, and 20.5% of dry weight, respectively) than those observed in our study by applying the same hydrolysis conditions. This might be due to the fact that biomass was air dried before the acid hydrolysis process in their study, and we used freeze-dried biomass. Our results align well with those of Visca et al. [63], who compared two drying methods before extracting *Scenedesmus* sp. and *Chlorella* sp. biomass: (1) cells were dried at 105ºC for 12 h, and (2) cells were freeze-dried, and then both were subjected to similar hydrolysis conditions as those described in the present work. *Scenedesmus* sp. yielded 30.5% carbohydrates and reached a maximum carbohydrate purity when freeze-drying pretreatment was used and lipids were removed (58.7% and 51.8%, depending on the extracting solvent). The authors concluded that *Chlorella* sp. carbohydrate content (17.7%) was not affected by the freeze-drying process because its cell wall is weaker. However, Safi et al. [11] reported that *Chlorella* sp. has a robust cell wall, and its disruption is a necessary preliminary step to quantify the total/maximum content of each metabolite. These findings agree with those reported by Stirk et al. [64], who observed that a simple freeze-drying step is not enough to break the tough cell wall of *Chlorella* sp., which requires methods combining freeze-drying with sonication or ball-milling. 

Carbohydrate contents evaluated without methods involving previous acid hydrolysis in 12 species of seaweeds washed with tap water and air dried on blotting paper to remove excess water ranged from 20.47 to 23.9% carbohydrates [65]. Our results obtained without acid pretreatment showed higher contents than those described by Manivannan et al. [65]: *C. saccharophilum, P. boryanum*, and *C. reticulata* yielded 39.6, 43.2, and 49.3% carbohydrates, respectively. These results agree with the total carbohydrate content quantified in microalgae *Neochloris oleoabundans* (∼40%) by Suarez Garcia et al. [66]. 

Further analytical studies of oligosaccharide structure and composition in the samples of novel microalgae, including the strains analysed in the present study, are necessary to unlock the potential applications of these microalgae and their components. In particular, algal polysaccharides have shown numerous industrial applications including antioxidant and antitumor effects, immunostimulating functions, cosmetics and cosmeceuticals, or prebiotic properties as functional foods or nutraceuticals [67,68].

## 4. Materials and Methods

### 4.1. Chemicals

Methanol (HPLC gradient grade) and tetrachloroethylene (synthesis grade) were purchased from Scharlab (Barcelona, Spain). Triethylamine (analysis quality) and phenylisothiocyanate (PITC) of reagent grade were supplied by Panreac (Barcelona, Spain); 1,1-diphenyl-2-picrylhydrazyl (DPPH), butylated hydroxyanisole (BHA), butylated hydroxytoluene (BHT), and anthrone (reagent grade) were supplied by Sigma-Aldrich (St. Louis, MO, USA). Formic acid (synthesis grade) and amino acids (aspartic acid, glutamic acid, histidine, arginine, proline, valine, lysine, methionine, isoleucine, and phenylalanine) were provided by Merck (Darmstadt, Germany). Phenolic compounds were supplied as follows: gallic acid, protocatechuic acid, (−) epicatechin, ferulic acid, *p*-coumaric acid, vanillic acid, syringic acid, and (+) catechin by Sigma–Aldrich Chemie (Steinheim, Germany), and rutin and gentisic acid by Merck (Darmstadt, Germany). Ultrapure water was obtained from a Milli-Q system from Millipore (Bedford, MA, USA).

### 4.2. Algal Material

Three microalgae clonal strains of the Phylum Chlorophyta were selected and provided by the Culture Collection at the Spanish Bank of Algae (located in Taliarte; east coast of Gran Canaria): *Chloromonas. cf. reticulata* (BEA0990B; Order Chlamydomonadales)*, Pseudopediastrum boryanum* (BEA0190B; Order Sphaeropleales)*,* and *Chloroidium saccharophilum* (BEA0031B; Order Watanabeales). Strains were isolated from samples bioprospected in Gran Canaria (Canary Islands), except *C. saccharophilum*, which was collected in the Chihuahua region (Mexico). *C. reticulata* was obtained from a terrestrial habitat in the central countryside area of the island, and *P. boryanum* was collected from a moist rock curtain in the northwest. Cultures were scaled up to 2 L Erlenmeyer flasks under controlled conditions (temperature: 23 ± 2 °C; irradiance: <100 µmol photons m^−2^ s^−1^; and photoperiod 16:8 light:dark (L:D)) in BG-11 culture medium (pH adjusted to 7.4), and continuous aeration was supplied with CO_2_ pulses at a rate of 1 min per hour. Growth curves were followed, and samples were harvested at the final exponential growth phase. After centrifugation at 8000 rpm for 15 min, biomass samples were freeze dried (6.5 L Labconco, Kansas City, MO, USA) and kept in sealed vials in darkness before analysis.

### 4.3. Radical Scavenging Activity (RSA) Measurements

Freeze-dried microalgae biomass (25 mg) was mixed with methanol (1.5 mL) using a vortex (Vortex Ika Genius 3) for 20 min. The mixture was heated at 40 °C for 10 min and sonicated for 10 min (this step was performed twice). Then, the algal material was centrifuged for 10 min at 9000 rpm in a microcentrifuge (Thermo Scientific, Heraeus fresco 17) and removed by filtration. The filtrate was evaporated to dryness in a rotary vacuum evaporator, and the residue was dissolved in methanol (100 µL).

The RSA of samples was evaluated using the DPPH free radical assay described by Bondet et al. [69] with some modifications. Briefly, 25 µL of the samples and standards BHA and BHT (0.2 g L^−1^) was mixed with 975 µL of DPPH solution (0.1 mM). The obtained mixture was vortexed and incubated for 20 min at room temperature in darkness. The neutralization of DPPH radical leads a decrease in absorbance monitored against a methanol blank at 515 nm using a Shimadzu 1800 UV-Vis spectrophotometer. The radical inhibition percentage was calculated by application of the equation

RSA = 100 × (1 − Abs in the presence of sample/Abs in the absence of sample)
(1)

Measurements were taken in triplicate, and the results were averaged.

### 4.4. Phenolic Compounds Determination

The extraction of phenolic compounds was carried out as described in the above section (RSA measurements) by mixing 200 mg of biomass with 10 mL of methanol. Once the filtrate was evaporated, the dry residue was suspended in 5 mL of acidified water (pH 1.5). Then, solid phase extraction (SPE) was used following the procedure of Dvořáková et al. [70] with modifications. The cartridges (Chromabon Easy, Macherey-Nagel, 500 mg, particle size 93 µm) were conditioned by successive elution with 2 mL of water, 6 mL of methanol, and 2 mL of water. The suspension was passed through the cartridge, and the retained phenolics were eluted with acetone (6 mL) and evaporated to dryness in a rotary vacuum evaporator. Finally, the residue was dissolved in 200 µL of mobile phase, filtered through a 0.20 µm nylon syringe, and transferred to a vial. Three replicates were used for the quantification.

**Figure 3:**
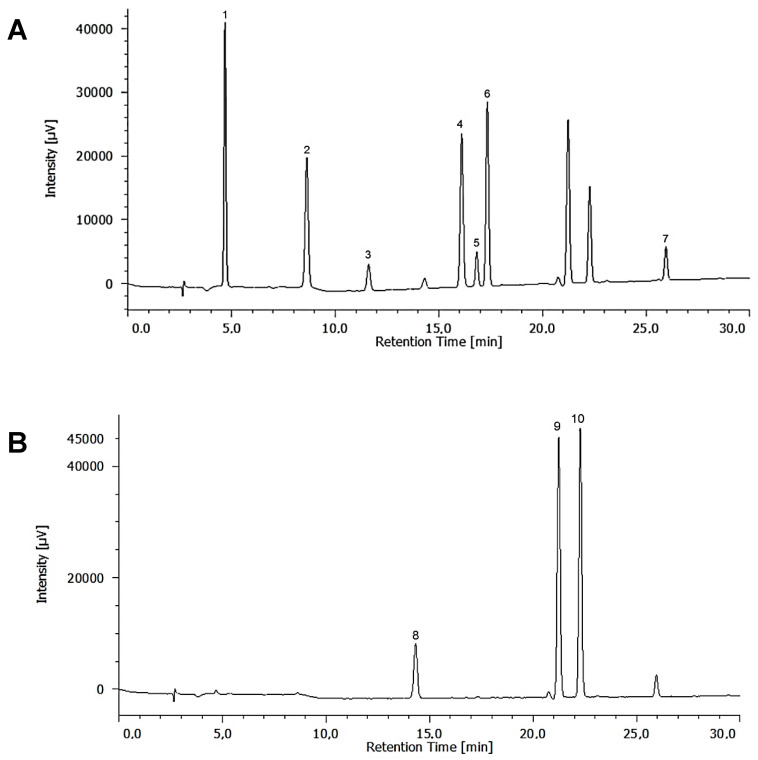
HPLC chromatograms of standard polyphenols: (**A**) 270 nm: 1, gallic acid; 2, protocatechuic acid; 3, catechin; 4, vanillic acid; 5, epicatechin; 6, syringic acid; 7, rutin. (**B**) 324 nm: 8, gentisic acid; 9, coumaric acid; 10, ferulic acid.

Chromatographic analysis was performed with a Jasco LC-4000 HPLC instrument equipped with a quaternary pump (PU-4180), an autosampler (AS-4150), photodiode array detector (MD-4015), and an LC-Net interface II. Data acquisition was carried out with ChromNav software. The phenolics were separated with a Varian C18 column (250 mm × 4.6 mm, 5 μm) and a guard column maintained at 30 °C. The gradient elution was performed using water with 0.1% formic acid as mobile phase A and methanol as mobile phase B with the following elution programme for eluent A: 0–5 min, 80% isocratic; 5–30 min, linear gradient from 80% to 40%. Finally, column was washed and reconditioned. Simultaneous monitoring was set at 270 nm (gallic acid, protocatechuic acid, catechin, vanillic acid, rutin, epicatechin, and syringic acid) and 324 nm (gentisic acid, coumaric acid, and ferulic acid) for quantification (Figure 3). Five different concentrations of each compound in the range of 1 to 50 mg L^−1^ were injected in triplicate. The presence of polyphenols in the extracts was confirmed by comparison of their retention times and overlaying of UV spectra with those of individual standard compounds.

### 4.5. Amino Acid Composition Determination

Extractions of amino acids were performed according to Machado et al. [47] with modifications. A total of 10 mg of freeze-dried biomass was mixed with 5 mL of deionized water for 2 min. The mixture was heated in a water bath at 70 °C for 30 min and was sonicated in an ultrasonic bath (Selecta, Spain) for 5 min followed by centrifugation for 10 min at room temperature (3000 rpm). The supernatant was collected and stored at −20 °C until analysis. Samples were examined under a microscope (Olympus BX40 model) to check complete cell wall lysis.

Protein hydrolysis for the total amino acid extraction was carried out as follows: HCl (6M, 2 mL) was added to 100 mg of freeze-dried microalgae in test tubes which were flushed under a N_2_ stream and heated in an oven at 110 °C for 24 h. Then, the mixture was neutralised by adding NaOH (6M), and deionized water was added up to 5 mL. The extracts were stored at −20 °C until analysis.

The amino acid derivatization procedure of Shi et al. [71] was modified by adding 5 mL of sample solution to 2.5 mL of PITC (1M in acetonitrile) and 2.5 mL of triethylamine (1M in acetonitrile). The resulting solution was stirred for 1 h at room temperature. Subsequently, 5 mL of tetrachloroethylene was added, the mixture was vigorously shaken, and the upper layer was collected. This step was performed twice, and the final solution was filtered through a 0.22 μm nylon syringe filter. Three replicates were used for the analysis.

**Figure 4:**
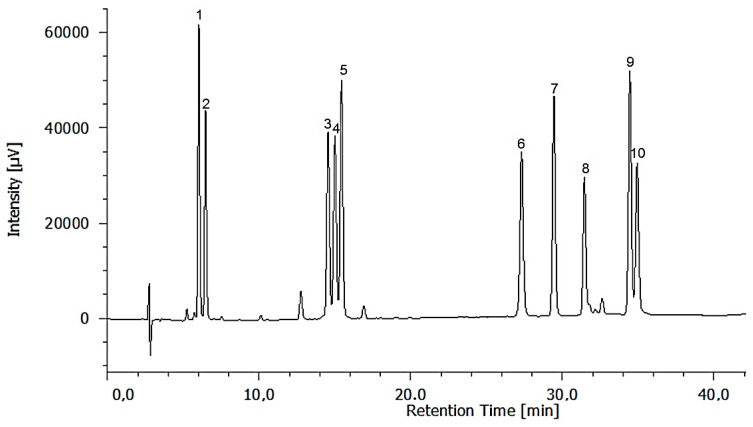
HPLC chromatogram of standard amino acids at 254 nm: 1, histidine; 2, arginine; 3, glutamic acid; 4, aspartic acid; 5, proline; 6, methionine; 7, valine; 8, lysine; 9, isoleucine; 10, phenylalanine.

Chromatographic analysis of six essential amino acids (histidine, methionine, valine, lysine, isoleucine, and phenylalanine) and four non-essential (arginine, proline and glutamic, and aspartic acids) was carried out with a Jasco LC-4000 HPLC instrument, as described above. The amino acid derivatives were separated with a Phenomenex C18 column (250 mm × 4.6 mm, 5 μm) and a Phenomenex guard column maintained at 30 ºC. The gradient elution was performed using water with 0.1% formic acid as mobile phase A and methanol as mobile phase B. The elution programme applied for eluent A was 0 min, 75%; 30 min, 40%; 40 min, 40%. Finally, column was washed and reconditioned. The flow rate was 1 mL min^−1^, and the injection volume was 10 μL. The calibration curves were made by plotting the integrated peak areas of the samples versus the concentration. Five different concentrations of each amino acid in the range of 1 to 40 mg L^−1^ were injected in triplicate. The presence of amino acids was confirmed by comparing their retention times with those of standard compounds (Figure 4).

### 4.6. Carbohydrates Quantification

Freeze-dried algal material was subjected to different pretreatments: (i) acid hydrolysis according to Templenton et al. [62], where the algal biomass (25 mg) was mixed with 250 µL of sulfuric acid (72 wt %) in a water bath at 30 °C for 1 h and then was heated at 121 ºC in sulfuric acid (4 wt %) in an autoclave (Micro 8, JP Selecta SA); (ii) aqueous extraction was carried out by stirring the biomass (25 mg) with 2.5 mL of ultrapure water during 1 h at room temperature according to Jansen [72]. Both extracts were centrifuged (3500 rpm for 10 min) and recovered by filtration.

Carbohydrate contents were determined using the colorimetric method described by Brooks et al. [73] with modifications. Anthrone reagent was prepared fresh daily by dissolving anthrone (200 mg) in 72% sulfuric acid (100 mL). This reagent (2 mL) was mixed with 1 mL of each sample (microalgae extracts and standard solutions), vortexed for 30 s, and heated for 10 min at 100 °C in a water bath. The test tubes were cooled in an ice bath for 5 min, and the absorbance was recorded at 505 nm on a Shimadzu UV-1800 spectrophotometer. A standard calibration curve was prepared with solutions of glucose in the range of concentrations from 20 to 200 µg mL^−1^. The results were expressed as grams of glucose equivalent (percentage of dry biomass). Three replicates were used for the determination of carbohydrate concentrations.

## 5. Conclusions

Two new simple, sensitive, accurate, and reproducible RP-HPLC methods were developed for detecting and quantifying 10 amino acids and 10 phenolic compounds in three novel selected microalgae strains. The antioxidant activities of extracts derived from *Chloromonas cf. reticulata*, *Psudopediastrum boryanum*, and *Chloroidium saccharophilum* determined in this study, as well as their amino acid, phenolic, and carbohydrate contents, confirm the potential possibilities of these microalgae species to be considered as novel source of bioactives for food, feed, and biomedical applications. Further research is needed to determine the impact of the growth conditions, illumination cycles during cell culture, their ability to accumulate metabolites, and, therefore, their specific potential.

## Figures and Tables

**Table 1:** Total polyphenol contents (µg g^−^^1^ of dry biomass) of the analysed microalgae strains.

Polyphenol	*Chloromonas cf. reticulata*	*Pseudopediastrum boryanum*	*Chloroidium saccharophilum*
Gallic acid	5.08 ± 0.25	1.44 ± 0.99	2.88 ± 1.22
Protocatechuic acid	n.d. *	1.23 ± 0.11	3.08 ± 1.34
Catechin	5.68 ± 0.74	4.14 ± 0.13	12.34 ± 1.13
Vanillic acid	1.61 ± 0.21	2.63 ± 0.23	14.15 ± 2.84
Epicatechin	10.43 ± 0.21	5.27 ± 0.84	1.90 ± 0.33
Syringic acid	3.12 ± 0.32	1.12 ± 0.10	5.84 ± 0.66
Rutin	n.d. *	6.40 ± 1.03	6.87 ± 3.42
Gentisic acid	n.d. *	n.d. *	2.42 ± 2.21
Coumaric acid	n.d. *	n.d. *	3.95 ± 2.09
Ferulic acid	n.d. *	2.44 ± 0.34	2.40 ± 1.67
Total	27.10 ± 2.03	26.40 ± 4.02	55.83 ± 16.90

* n.d.: not detected.

**Table 2:** Free analysed amino acid contents (µg g^−1^ of dry weight) of the three microalgae strains.

Amino Acid	*Chloromonas cf. reticulata*	*Pseudopediastrum boryanum*	*Chloroidium saccharophilum*
Arginine	107.88 ± 1.67	448.00 ± 90.00	668.34 ± 48.16
Glutamic acid	461.82 ± 1.70	1937.00 ± 28.00	5630.37 ± 135.89
Aspartic acid	89.31 ± 0.55	157.57 ± 16.49	805.95 ± 18.82
Proline	112.99 ± 0.51	1282.00 ± 38.00	5546.42 ± 141.14
Methionine	146.00 ± 6.30	201.00 ± 77.00	805.95 ± 90.52
Valine	130.00 ± 0.38	285.00 ± 6.14	1379.65 ± 159.98
Lysine	196.84 ± 0.87	697.65 ± 52.14	2847.93 ± 157.67
Isoleucine	65.34 ± 0.08	86.03 ± 4.00	866.81 ± 15.26
Phenylalanine	107.42 ± 0.19	108.00 ± 2.00	1379.65 ± 30.57
Histidine	116.67 ± 1.22	154.00 ± 21.00	530.92 ± 38.35
Sum of amino acids in µg g^−1^ of dry weight			
∑NEEA	772.0 ± 4.43	3824.6 ± 172.5	12,651.1 ± 344.0
∑EEA	762.3 ± 9.04	1531.7 ± 162.3	7810.9 ± 492.5
∑FAA	1534.33 ± 13.5	5356.3 ± 334.8	20,462.0 ± 836.5

∑NEEA, sum of non-essential amino acids; ∑EEA, sum of essential amino acids; ∑FAA, sum of free amino acids.

**Table 3:** Contents of 10 analysed amino acids (mg g^−^^1^ of dry weight) of three microalgae strains.

Amino Acid	*Chloromonas cf. reticulata*	*Pseudopediastrum boryanum*	*Chloroidium saccharophilum*
Arginine	21.29 ± 3.47	7.12 ± 0.75	5.98 ± 0.66
Glutamic acid	23.84 ± 6.25	19.48 ± 1.41	30.50 ± 1.28
Aspartic acid	14.67 ± 3.59	11.38 ± 0.84	3.44 ± 0.21
Proline	51.69 ± 1.75	49.90 ± 4.25	31.68 ± 1.42
Methionine	40.81 ± 9.78	137.20 ± 24.41	29.89 ± 8.96
Valine	19.72 ± 1.79	20.21 ± 1.59	18.48 ± 0.45
Lysine	31.56 ± 1.66	36.20 ± 2.32	36.49 ± 1.99
Isoleucine	13.28 ± 1.35	13.43 ± 1.32	11.36 ± 0.71
Phenylalanine	19.76 ± 1.37	20.01 ± 1.20	14.92 ± 0.21
Histidine	6.07 ± 0.13	5.63 ± 0.69	1.81 ± 0.01
Sum of amino acids in mg g^−1^ of dry weight (%)			
∑NEEA	111.5 ± 15.1 (45.9)	87.9 ± 7.25 (27.4)	71.6 ± 3.57 (38.8)
∑EEA	131.2 ± 16.1 (54.1)	232.7 ± 30 (72.6)	112.9 ± 11.3 (61.2)
∑TAA	242.7 ± 31.1	320. 6 ± 38.1	184.5 ± 14.9

∑NEEA, sum of non-essential amino acids; ∑EEA, sum of essential amino acids; ∑TAA, sum of total amino acids.

## Data Availability

The data used to support the findings of this study are included in the article.
